# Nickel-doped lanthanum cerate nanomaterials as highly active electrocatalysts

**DOI:** 10.3389/fchem.2022.1064906

**Published:** 2022-11-22

**Authors:** Tehmeena Maryum Butt, Safia Erum, Ayesha Mujtaba, Dmitry Medvedev, Naveed Kausar Janjua

**Affiliations:** ^1^ Department of Chemistry, Quaid-i-Azam University, Islamabad, Pakistan; ^2^ Laboratory of Electrochemical Devices based on Solid Oxide Proton Electrolytes, Institute of High Temperature Electrochemistry, Yekaterinburg, Russia; ^3^ Ural Federal University, Yekaterinburg, Russia

**Keywords:** Ni-doped lanthanum cerates, electrocatalysis, high-temperature impedance spectroscopy, oxygen evolution reaction, cyclic voltammetry Ni-doped lanthanum cerates, cyclic voltammetry

## Abstract

The efficient oxygen evolution reaction (OER) and oxygen reduction reaction (ORR) catalyst materials are crucial in the energy research domain due to their tunability. Structural modification in perovskites such as lanthanum cerates (LaCeO_3_) upon doping at A or B sites significantly affects the surface activity and enhances the catalysis efficacy. Herein, B-site nickel-doped lanthanum cerate (LaCe_1-x_Ni_x_O_3±δ_) nanopowders were applied as ORR indicators in high-temperature electrochemical impedance spectroscopy for solid-oxide fuel cell (SOFC) tests and in cyclic voltammetric OER investigations in alkaline medium. The integration into SOFC applications, *via* solid-state EIS in a co-pressed three-layered cell with LCNiO as cathode, is investigated in an oxygen–methane environment and reveals augmented conductivity with temperatures of 700–850°C. The higher electrokinetic parameters—including diffusion coefficients, D_o_ heterogeneous rate constant, k_o_, and peak current density for OER in KOH-methanol at a LCNiO-9-modified glassy carbon electrode—serve as robust gauges of catalytic performance. CV indicators and EIS conductivities of LaCe_1-x_Ni_x_O_3±δ_ nanomaterials indicate promising potencies for electrocatalytic energy applications.

## Introduction

Increasing environmental concerns and energy crises have motivated researchers to develop new and sustainable energy resources, as well as corresponding energy conversion and storage devices such as fuel cells, electrolyzers, and batteries (Zhang et al., 2019). The efficiency of these energy devices mostly relies on the electrocatalytic reactions of water in different media. Among various reactions in water/oxygen electrochemistry (Zaman et al., 2021; Xu et al., 2022), the oxygen reduction reaction (ORR) and oxygen evolution reaction (OER) play pivotal roles in many energy devices. These reactions are considered to be crucial parameters that affect the efficiency of many energy-conversion and storage devices (Wang et al., 2020a; Ji et al., 2022; Yang et al., 2022).

OER is the anodic reaction in electrolyzers and metal–air batteries. Hydrolysis of water *via* the electrochemical (EC) pathway to produce high-purity hydrogen (hydrogen evolution reaction; HER) is a sustainable and cost-effective approach toward meeting the world’s clean energy demand (Rossmeisl et al., 2005; Zhou et al., 2018; Asim et al., 2022). Electrochemical water splitting is comprised of two half-reactions, an anodic reaction in which oxygen is produced (OER) and a cathodic reaction in which hydrogen is produced (HER). The water-splitting route suffers substantial overpotential losses, especially at the anode (Reier et al., 2012; Butt et al., 2020; Butt et al., 2022). OER is a multiple-electron transfer process that occurs at the anode. This pH-dependent process requires a potential difference of 1.23 V (*vs.* reversible hydrogen electrode; RHE) and is kinetically obstructed by the accumulation of energy at each step (Tahir et al., 2017; Badruzzaman et al., 2020; Zhou and Fan, 2020). To enhance the overall efficiency of the OER process, various functional materials (FMs) are being designed to offer catalytic propensities at the electrolyte interfaces with the cathode and anode. Electrocatalysts reduce the OER overpotential and upsurge the efficiency of an FM-based EC system. In this regard, there is always a need to discover efficient potential water-oxidation electrocatalysts (WOECs) (Reier et al., 2012; Wang et al., 2020b).

In the field of electrochemical energy generation, nanostructured perovskite oxides have attracted much attention due to their low cost and electrocatalytic properties (Rincón et al., 2014; Li et al., 2016; Khan et al., 2020; Liu et al., 2021). Lanthanum cerates (LCO) have a fluorite-type structure with high-temperature phase stability and a high thermal expansion coefficient (Huang et al., 2016; Singh et al., 2016). The enhanced ionic conductivity of lanthanum cerates makes them promising candidates for electrochemical applications. Replacement of cerium in the lanthanum cerate structure at the B site is a vital factor in improving the conductivity and catalytic potential of these perovskites (Butt et al., 2020; Liu et al., 2021).

The present work is devoted to understanding the structural and redox properties of selected compositions of nickel-doped lanthanum cerates. Herein, nickel-doped lanthanum cerates (LaCe_1-x_Ni_x_O_3±δ_; 0.01 ≤ x ≤ 0.09) were synthesized and subjected to rigorous characterization—using the techniques of XRD, FTIR, scanning electron microscopy (SEM), energy-dispersive X-rays (EDX), and TGA analysis—and investigated to their full extent in SOFC and OER studies. LCNiOs are reported as potential, cost-effective electrocatalysts to mitigate overpotential losses in the path of the OER process occurring at the anode. The EC inferences from CV and EIS studies have helped to integrate LCNiOs in SOFC applications to assess the potential of synthesized nanomaterials for intermediate/high-temperature electrochemical devices. In solid oxide fuel cells (SOFCs), their electrochemical progression with temperature has been thoroughly investigated. A fabricated co-pressed three-layered asymmetrical cell (LCNiO as cathode, 10 mol% gadolinium-doped ceria, 10GDC as an electrolyte, and nickel oxide, NiO as the anode material) was investigated in oxygen at the cathode side and methane at the anode side (oxygen–methane environment) by high-temperature impedance spectroscopy (HT-EIS). AC conductivities were evaluated and were observed to enhance the temperature rise from 700 to 850 **°**C. The synthesized nano-perovskites exhibited enhanced electrocatalytic performance, higher stability for the OER, and nominal performance in fuel cell mode, and these findings are reported here for the first time.

## Experimental

### Chemicals and reagents

Reagents included lanthanum nitrate hexahydrate (La(NO_3_)_3_·6H_2_O), cerium nitrate hexahydrate (Ce(NO_3_)_3_·6H_2_O), nickel nitrate hexahydrate (Ni(NO_3_)_2_·6H_2_O), ammonium hydroxide (NH_4_OH), absolute ethanol, potassium hydroxide (KOH), methanol, potassium ferrocyanide trihydrate (K_4_[Fe(CN)_6_]·3H_2_O), Nafion^®^, and freshly prepared deionized water. All chemicals were purchased from Sigma Aldrich (99.9%) and used without any purification. For impedance studies, nickel oxide (NiO) was used as an anode material and commercially synthesized gadolinium-doped ceria (Ce_0.9_Gd_0.1_O_2–δ_, 10GDC) was used as an electrolyte; both were purchased from Fiaxell Technologies, Switzerland, and used without additional treatment.

### Powder preparation

**FIGURE 1:**
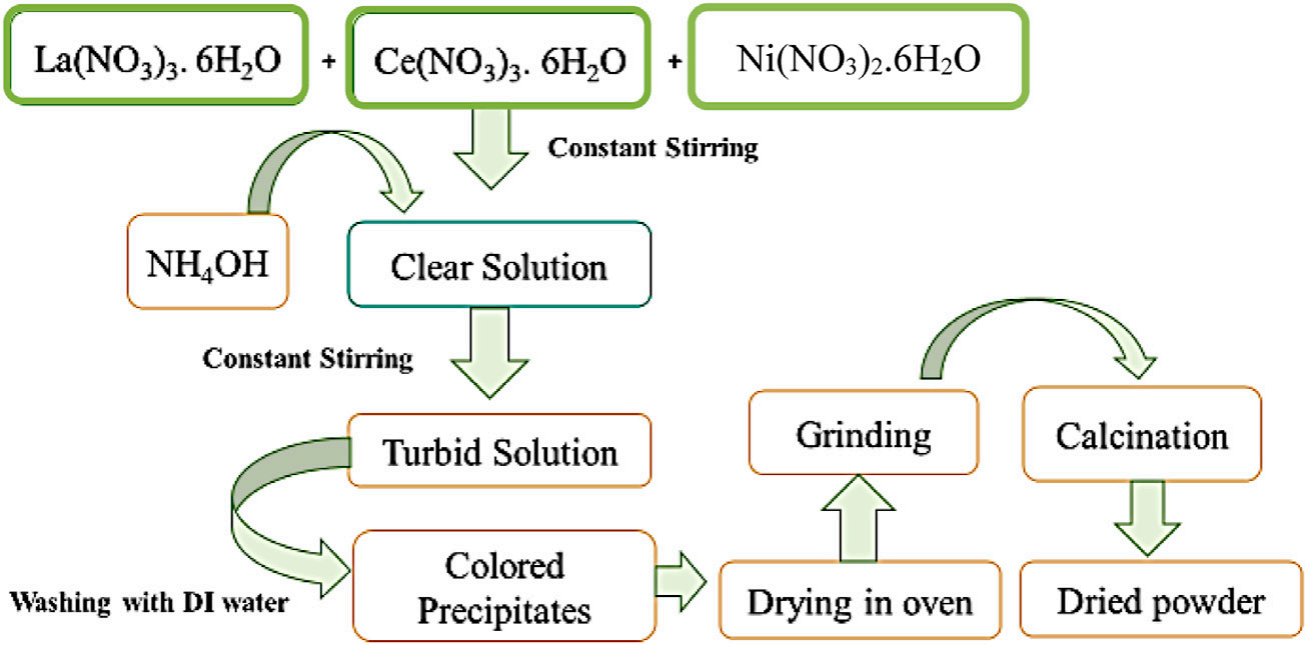
Flowsheet diagram for the synthesis of pure and nickel-doped LCO.

Powders of pure and nickel-doped lanthanum cerate were prepared using a precipitation method (Butt et al., 2020). Nitrate precursors of salts were taken in a proper molar ratio and mixed separately in deionized water with constant stirring until a clear solution was obtained. Ammonia was added, and precipitates were collected at a pH range of 9–11. The precipitates were heated at 80°C to evaporate the solvent. The obtained thick slurry of precipitates was dried in an oven at 110°C for 9–10 h and calcined at 500°C. The calcined product was ball-milled (ground) using acetone for 5 h for homogeneous mixing (Singh et al., 2016). A flowsheet diagram for the synthesis of nickel-doped lanthanum cerate is presented in Figure 1. The Ni-doped lanthanum cerate (LaCe_1-x_Ni_x_O_3±δ_; LCNiO), was prepared in three compositions with nickel contents (x) of 0.01, 0.05, and 0.09; details of compositions with sample codes and molecular formulae are provided in Supplementary Table S1.

### Material synthesis for HT-EIS studies

To explore the capability of LCNiO nanomaterials as cathodes for SOFCs, high-temperature impedance (HT-EIS) measurements were carried out. A complete cell consisting of anode, cathode, and electrolyte material was fabricated. Nickel oxide, which was prepared by a simple co-precipitation method (Deshpande, 2016), was used as anode material. Ammonia was added dropwise to nickel nitrate solution to precipitate out a greenish nickel hydroxide gel. The resultant gel was oven-dried at 110°C and heated at 350°C for 3 h to obtain the corresponding nickel oxide powder. The resultant product was ball-milled to grind the NiO powder to homogeneity. Hence, a greyish-black nickel oxide was obtained, which was used as anode material during the cell’s fabrication for high-temperature impedance studies.

### Fabrication of asymmetrical cells

A tri-layered cell (20 mm diameter and 1 mm thickness) was prepared by loose pressing under uniaxial pressure in a 20 mm die, with 0.35 g of each anode and cathode material, while 0.25 g electrolyte was sandwiched between the anode and cathode layers. Nickel-doped lanthanum cerate was used as a cathode material, with gadolinium-doped ceria as an electrolyte and nickel oxide as an anode material. The asymmetric cell was placed directly in an open-flange fuel cell tester with gold mesh as the cathode-current collector and Ni mesh as the anode-current collector. EIS testing was conducted at 973, 1023, 1073, and 1123 K using a Gamry potentiostat (Raza et al., 2010).

### Microstructure characterization

Nickel-doped lanthanum cerate materials were synthesized and characterized by various techniques to ensure their successful synthesis and purity. The crystal structure of LCNiO was analyzed by XRD by PANalytical Xpert highscore, using CuKα_1_ with a wavelength of 1.54 nm and a scan range of 20°–80°. The ceramics’ microstructures were observed by SEM, and their elemental compositions were confirmed by EDX. SEM and EDX were carried out using a TESCAN scanning electron microscope. Thermogravimetric (TGA) curves were acquired from a Shimadzu TGA/DTA thermal analyzer in air.

### Electrochemical characterization

Electrochemical studies were performed to investigate the redox potential of nickel-doped lanthanum cerate nanoceramics for the OER. For this purpose, a glassy carbon (GC) electrode with geometric area of 0.07 cm^2^ was modified with the synthesized nanocatalysts and employed as a working electrode. The electrocatalyst-modified electrode was prepared using a drop cast method. The surface of GC was wetted with 2 μL ethanol, and 0.1 mg of catalyst was dropped on the working surface followed by drop-casting of 2 μL of 0.5% V/V Nafion solution (Mujtaba and Janjua, 2016; Butt et al., 2020). The dried electrode (LCNiO/GC) was used to acquire the electrochemical data. Each time, the GC electrode was subjected to the preconditioning measures of polishing and ultrasonication for improved performance.

Potassium ferrocyanide is a standard analyte for examining the conductive behavior of electroactive materials, including electrocatalysts. The conductive nature of LCNiO materials was observed using cyclic voltammetry and electrochemical impedance spectra in the presence of 5 mM potassium ferrocyanide in 1 M KCl, using the potentiostat/galvanostat interface 1000 by Gamry. A three-electrode cell assembly was used for electroanalytic measurements. The catalyst modified GC as a working electrode and calibrated Ag/AgCl (3 M KCl) as a reference electrode (RE), and platinum wire was used as a counter electrode (CE). The response of the modified electrode toward water oxidation was studied in 1 M KOH and methanol as a facilitator (Guzman et al., 2013). Voltammetric investigations were performed in the potential range of 0.2–1.8 V (versus RHE) in argon- and oxygen-saturated environments separately, while EIS and chronoamperometry were performed at the potential at which current density approaches 10 mA cm^−2^.

HT-EIS measurements in the solid state were carried out by coupling a potentiostat with an open-flange fuel cell tester, and readings were taken at 973, 1023, 1073, and 1123 K. All measurements were carried out with an initial frequency of 1 MHz and final frequency of 0.1 Hz.

## Results and discussion

### Morphological characterization

**FIGURE 2:**
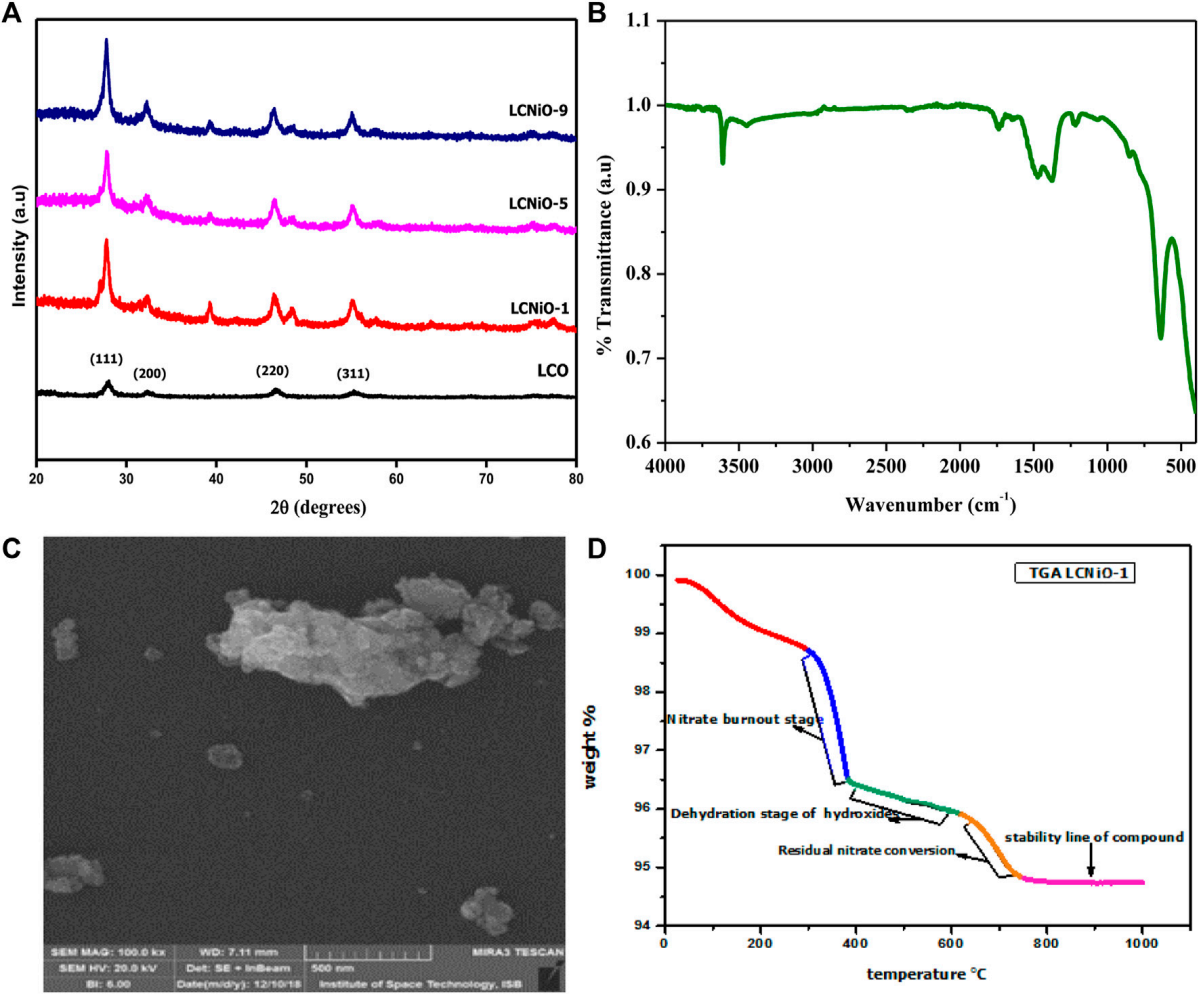
**(A)** An overlay of XRD patterns of pure and nickel-doped lanthanum cerates; LCNiO-1 to LCNiO-9, **(B)** FTIR spectrum for LCNiO-9, **(C)** SEM micrograph of LCNiO-9, and **(D)** TGA plot for LCNiO-1.

XRD diffractograms of lanthanum cerate (LCO) and nickel-doped lanthanum cerate (LCNiO) series are presented, showing that single-phase morphology was maintained in nickel-doped lanthanum cerate materials. The result obtained for pure LCO is presented in the inset of Figure 2A and matches well with the reference card of lanthanum cerate perovskite materials with cubic symmetry (JCPDS 01-080-5546). The XRD patterns of synthesized nickel-doped lanthanum cerate ceramics with various amounts of the dopant, from x = 0.01 (LCNiO-1) to 0.09 (LCNiO-9), are shown together in Figure 2. These patterns demonstrate no extra peaks for impurity and a slight peak shift with increased intensities, verifying the successful incorporation of nickel dopant into the lanthanum cerate crystal matrix. An additional peak at a 2θ value of 40° was observed for LCNiO, in comparison to the LCO pattern, which corresponds to the assimilation of dopant into the perovskite structure.

All the compositions showed consistency in diffractograms, with the only difference lying in the intensity, which increased as the dopant concentration increased. Peak intensities in the XRD pattern of LCNiO-9 were increased in comparison to LCNiO-1 and LCNiO-5; therefore, LaCe_0.91_Ni_0.09_O_3±δ_ is the optimum composition for the tolerance of the doping concentration among those considered. The crystal structure of nickel-doped lanthanum cerates was found to be cubic, with a tolerance factor range of 0.813–0.825 (ideally, it is unity). The crystallite size (D) was calculated using the Debye–Scherrer equation (Monshi et al., 2012):

D=kλ/β⁡cos⁡θ (1)

where k is the constant, λ is the wavelength of X-rays used, and *ß* is the full width at the half-maximum of the peak. The crystallite size for pure lanthanum cerate was 11 nm, which is in the range of 18–28 nm for doped materials (Supplementary Table S2). The increased crystallite sizes of doped compositions compared to their parent material is further evidence of the successful incorporation of nickel ions into lanthanum cerates. This upsurge in particle size upon dopant addition can be attributed to the oxygen vacancies produced to compensate for the charge imbalance; this provides a path toward greater oxygen ion movement and increased grain growth (Mondal et al., 2015; Sankannavar et al., 2018). The crystallite size of LCNiO-9 showed the lowest value (18 nm) among all compositions, which revealed a better electrocatalytic property of this composition for OER (Yang et al., 2019).

FT-IR is used to determine the phase purity and any impurity present in the sample. For doped lanthanum cerates, FTIR spectra in the selected range (4000 cm^−1^ to 400 cm^−1^) were recorded in powder form, and spectra of LCNiO-9 are presented in Figure 2B. For metal oxide vibrations, vibrational bands were observed in the range of 400–700 cm^−1^ (Cheng et al., 2004; Singh et al., 2016), which is due to the internal stretching motion of the Ni ions against oxygen. The vibrational band observed at about 3609 cm^−1^ is for O-H stretching, which could be due to hydroxyl groups in the surface-adsorbed water. The vibrational peaks correspond to C-O vibration bands for tridentate carbonates observed at about 1407 and 1371 cm^−1^ (Liao et al., 2002), which are due to adsorbed CO_2_. The entire spectrum shows a sharp peak for the metal oxide band and a weak vibrational peak of impurity, confirming the successful formation of perovskite.

SEM is widely used for analyzing the surface morphology of materials. It provides information about the particle shape of the material under analysis, as well as the homogeneity and non-homogeneity of a phase formed on the surface (Echlin, 2013). SEM micrographs of the synthesized samples are presented in Supplementary Figure S1. Small irregular nanoflakes of LCNiO are visible, with an anticipated average particle size of 20–40 nm. The soft, spongy, and sticky nature of ceria particles may result in the agglomeration of nanoparticles (Masui et al., 2002). The stoichiometry of elements in LCNiO was confirmed by EDX analysis. The EDX spectrum of LCNiO-9 is presented in Figure 2C. EDX peaks corresponding to La, Ce, O, and Ni were observed in all spectra, and elemental analysis inferred the stoichiometry of the nanomaterials (Supplementary Table S3).

Thermogravimetric analysis was performed to check the thermal stability of compositions over a temperature range. Thermogravimetric data obtained for LCNiO-1 is presented in the form of a plot (Figure 2D). Initial mass loss was observed due to the removal of adsorbed water, represented by the red region below 300°C. The slope at 330°C (blue region) can be ascribed to the decomposition and burnout of most of the nitrates of lanthanum, cerium, and nickel into precursor powders. The second weight loss between 400 and 600°C (green region) is mostly due to the dehydration of lanthanum, cerium, and nickel hydroxides. A small weight loss at 600–700°C (orange region) is due to residual nitrate conversion and intermediate decomposition, while the straight line (pink region) represents the formation and stability of the requisite compound (Djoudi et al., 2012).

### Electrochemical characterization

#### The electrochemical active surface area of modified electrodes

As previously explained, electrochemical data was acquired using catalyst-modified GCE, and all electrodes were characterized using cyclic voltammetry. The electrochemical active surface area of the modified electrodes was determined using the Randles–Sevcik equation for a reversible system (Mujtaba and Janjua, 2016):

$${Ip=2.69\times 10}^{5}n^{3/2}{AD}_{o}^{1/2}\upsilon^{1/2}C$$ (2)

where I_p_ is the redox peak current of potassium ferrocyanide, n is the number of electrons transferred, A is the active surface area, D_o_ is the diffusion coefficient (0.76 × 10^–5^ cm^2^ s^−1^, 298 K), υ is the scan rate (0.1 Vs^-1^), and C is the analyte concentration (5 mM). Estimated active surface areas of modified electrodes are observed in the range of 0.01–0.06 cm^2^, as tabulated in Supplementary Table S2. The highest active surface area was observed for LCNiO-9, as well as the best conductive responses with the highest peak current for the redox couple Fe^2+^/Fe^3+^ in the reference analyte K_4_[Fe(CN)]_6_. The cyclic voltammetric response of LCNiO-9 for the Fe^2+^/Fe^3+^ redox couple is presented in Supplementary Figure S2A.

#### Electrochemical impedance spectroscopy

The electron transfer capacity of all modified electrodes was inspected via EIS with a Fe^2+^/Fe^3+^ system in 1 M KCl. The impedance spectra are shown in the form of a Nyquist plot in Supplementary Figure S2B for one of the representatives (LCNiO-9) of the LCNiO series. The small semicircular part of the plot at higher frequencies signifies electron transfer resistance (R_ct_) and is representative of the kinetics of the redox system. The diameter of the semicircle is dependent on the characteristics of the electrode. The inclined part at lower frequencies corresponds to Warburg resistance (R_w_), indicating the diffusion of electroactive species at the interface of electrode and electrolyte (Heiduschka et al., 1994).

The experimental data were fitted to an equivalent circuit model (given in the inset of Supplementary Figure S2B) using Gamry software, and the EC parameters retrieved therein are given in Table 1. The solution resistance, R_s_, and Warburg resistance, R_w_, vary slightly, as these are characteristic properties of the electrolyte and the diffusion of electroactive species toward the modified electrode, respectively. Although the concentration of the electrolyte is maintained, R_s_ and R_w_ values may vary due to changes in the intrinsic nature of the interfacial region with the dopant concentration. However, R_ct_ and constant phase element (CPE), which is utilized to account for the heterogeneous surfaces of the coated electrodes, are dependent upon the conductive nature of the material and consequently vary for all modified electrodes. The modified electrodes showed surface roughness in the range of 0.85–0.95. The apparent electron transfer rate constant (k_app_) for OER on all the modified electrodes was calculated using the following equation (Sankannavar et al., 2018; Yang et al., 2022):

$$k_{app}{=RT/F}^{2}R_{ct}C$$ (3)

where k_app_ is a measure of the ease of electron-transfer kinetics across the electrode–electrolyte interface and increases with a decrease in electron transfer resistance. The extracted impedance parameters and the apparent electron transfer rate constants for OER on all the modified electrodes are presented in Table 1.

**TABLE 1:** Data derived from equivalent circuit simulations of EIS spectra.

Sample	R_ct_ (kΩ)	CPE (µF)	R_w_ (µΩ)	R_s_ (kΩ)	k_app x_ 10^–3^ (cms^−1^)
Codes					
LCNiO-1	50.90	4.49	10.27	3.05	1.04
LCNiO-5	35.01	5.23	26.58	0.57	1.51
LCNiO-9	28.92	4.62	230.7	0.43	1.83

Various kinetic performance parameters, as extracted from the EC testing, including diffusion coefficient and apparent rate constant, correlate with the material properties, and their potencies for bulk catalysis are envisioned using milli- to micromolar amounts of the materials. In the present study, the R_ct_ value is also inferred to correlate with compositional variation; it decreases in the order LCNiO-1> LCNiO-5>LCNiO-9 and is lowest for LCNiO-9, suggesting it to be a better catalytic material for electrochemical applications.

### SOFC measurements in a solid-state three-layered device

**FIGURE 3:**
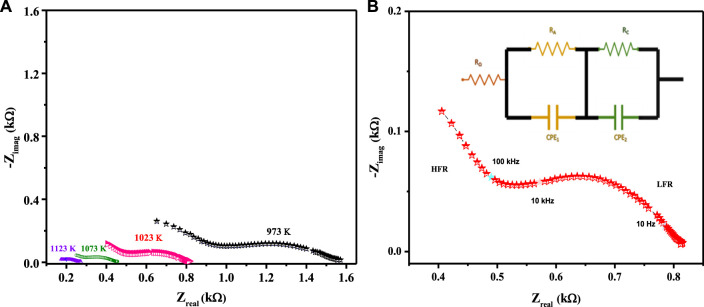
HT-EIS spectra of three-layered co-pressed asymmetrical cell with 3L-LCNiO-5 in oxygen-methane atmosphere at **(A)** 973, 1023, 1073, 1123 K and **(B)** A potentiostatic orthonormal EIS spectrum of 3L-LCNiO-5 based cell recorded at 1023 K, EIS model used for simulation of experimental data in the inset.

The electrocatalytic potency of a cathode is a function of its oxygen diffusion rate, and the LCNiOs were thus further investigated via solid-state high-temperature impedance spectroscopy (HT-EIS). Ni-doped lanthanum cerate (LaCe_1-x_Ni_x_O_3±δ_; x = 0.01, 0.05, and 0.09) nanoceramics were tested as SOFC cathodes, with 10GDC (10 mol% gadolinium-doped ceria) as an electrolyte and nickel oxide (NiO) as anode material in a three-layered co-pressed device. This three-layered cell was placed in the open-flange fuel cell tester, and high-temperature EIS testing was carried out at 973, 1023, 1073, and 1123 K using a highly precise thermocomputer device for pre-setting and controlling temperature profiles. EIS spectra observed at different temperatures for the three-layered 3L-LCNiO-3 device are presented in Figure 3A.

An oxidant is reduced at the cathode, the fuel is oxidized at the anode in SOFCs, and different processes may occur at the electrode/electrolyte interface. These processes may include diffusion, adsorption, charge transfer reactions, and many others (Ding et al., 2019), which can be studied using different resistance values obtained from EIS data at higher temperatures.

Usually, the electrochemical performance of a fuel cell is evaluated by the ohmic, activation, and concentration polarizations/drops. In such an EIS spectrum, the intercept with the Z_real_ axis at high frequency provides ohmic resistance (R_o_), which is mainly contributed by electrolyte, wires, and current collectors. The intercept with the Z_real_ axis at low frequency provides total resistance (R_t_). Polarization resistance (R_p_) is the difference between low- and high-frequency intercepts at the Z_real_ axis, which are mainly due to charge transfer and diffusion processes (Ding et al., 2011; Mroziński et al., 2019; Farsani et al., 2020).

**FIGURE 4:**
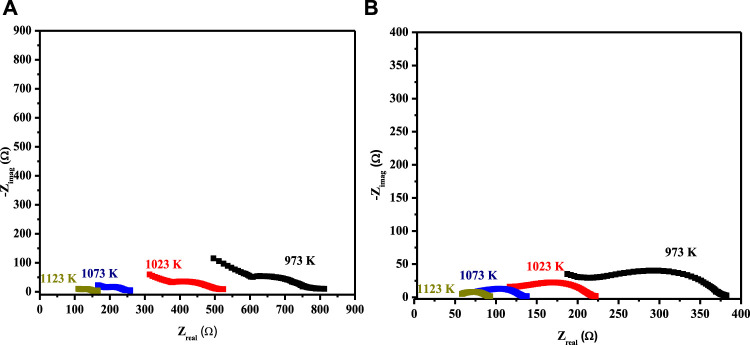
HT-EIS square-scaled spectra of three layered co-pressed asymmetrical cell with 3L-LCNiO-1 **(A)** and LCNiO-9 **(B)** in oxygen-methane environment at 973, 1023, 1073 and 1123 K.

The EIS spectrum for the three-layered cell is presented in Figure 3B, with an equivalent circuit model adopted for the simulation of data in the inset. The EIS spectrum exhibits two overlapped semicircular arcs, as presented in Figure 3A. A small semicircle at the low-frequency region corresponds to the charge transfer process at the electrode/electrolyte interface; however, the other semicircle at the high-frequency region corresponds to diffusion processes at the electrode for the oxygen-exchange reactions at the surface. The low-frequency arc corresponds with the same pattern upon temperature variation; however, a very slight change in the high-frequency arc was observed, owing to its temperature-dependent nature (Mohammadi et al., 2014). The HT-EIS spectra show an explicit alleviation of ohmic and polarization resistances with a temperature rise from 973 to 1123 K. This behavior can be attributed to increased charge transfer conductivity, corresponding to interfacial capacitance and/or ionic conductivity. Figures 4A,B depict the similar EIS experimentation for 3L-LCNiO-1 and 3L-LCNiO-9 respectively, at various temperatures (973, 1023, 1073, and 1123 K) in an oxygen–methane environment.

All EIS spectra were model-fitted to obtain the value of resistances (ohmic and polarization) and various electrical parameters like resistivity/conductivity, and activation energies were also estimated (Georgieva et al., 2017). The magnitude of resistances against ion movement was calculated using the simulations, and data were extracted by fitting the EIS spectra at each temperature (Table 2).

**TABLE 2:** Different resistances calculated from simulation of HT-EIS data for three-layered cell based on LCNiO-1, LCNiO-5, and LCNiO-9.

Temperature (°C)	R_o_ (Ω)	R_p_ (Ω)	R_t_ (Ω)
	**LCNiO-1**		
	700	141	239	98
750	100	321	221
800	68.07	74.28	6.2
850	69.35	114.50	45.1
	**LCNiO-5**		
	700	156	1570	1414
750	301	1342	1040
800	40	387	346
850	6.2	244	237
	**LCNiO-9**		
	700	90	1144	1054
750	484	190	294
800	342	268	74
850	42	205	163

**FIGURE 5:**
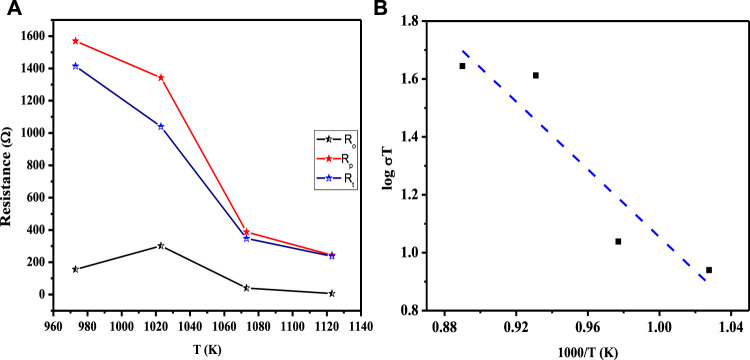
**(A)** Functional plot between various resistances (R_o_, R_p_, and R_t_) and working temperatures of the asymmetrical cell using LCNiO-5; **(B)** Arrhenius plot for the estimation of activation energy in LCNiO-5 based cell.

The decreasing trend of polarization resistance of the 3L-LCNiO-5-based cell with temperature rise is presented in Figure 5A and shows an increase in the ionic conduction of the composited device at higher temperatures (Nasir et al., 2020). From the observed values of polarization resistances, the conductivity was observed to also increase rapidly from the value at 973 K through 1023 K, while after 1073 K, there was no significant change in conductivity value (Prakash et al., 2017). It can be inferred that 1073 K is the optimum temperature for these materials to work best, with low resistivity and good conductivity, under ambient conditions. The Arrhenius equation gives a clear relationship between the conductivity of the solid, or other EC devices, and temperature. Activation energy (E_a_) is a major barrier in solid-state devices, and electrocatalysts are required to lower its value by adopting an alternative energy conversion path. In the cathodic performance of the Ni-doped lanthanum cerates in the 3-L configuration, the activation energy was estimated from the temperature-dependent EIS data using the Arrhenius equation:

$$\ln\sigma T=\ln\;\sigma_{o}-Ea/RT$$ (4)

The activation energy for ionic conduction was calculated for the composited devices from the slopes of the Arrhenius plots (-E_a_/R) (Nasir et al., 2020), as presented in Figure 5B, to be 2.99 eV for LCNiO-1, 1.76 eV for LCNiO-5, and 1.16 eV for LCNiO-9. Electrical bandgaps were calculated using conductivity measurements (electrical bandgap = 2E_a_). The electrical bandgap values of LCNiO-1, LCNiO-5, and LCNiO-9 are 5.98, 3.52, and 2.32 eV, respectively, which conform to the better tuning of the LCNiO-9 composition. The low activation energy and bandgap values illustrate the superior conductive nature of LCNiO-9 and the facile oxygen reduction process at the electrode surface, which also corresponds well to the results obtained by electrochemical analysis in the OER.

### Oxygen evolution reaction catalytic activity

Voltammetric experiments were carried out to analyze the performance of the synthesized nanomaterials in the OER in basic medium (electrolyte; 1 M KOH), in the optimal potential scan range of 0.2–1.8 V. In this potential range, a distinct anodic peak was observed, which could be ascribed to water electrolytic oxidation or the OER peak.

**FIGURE 6:**
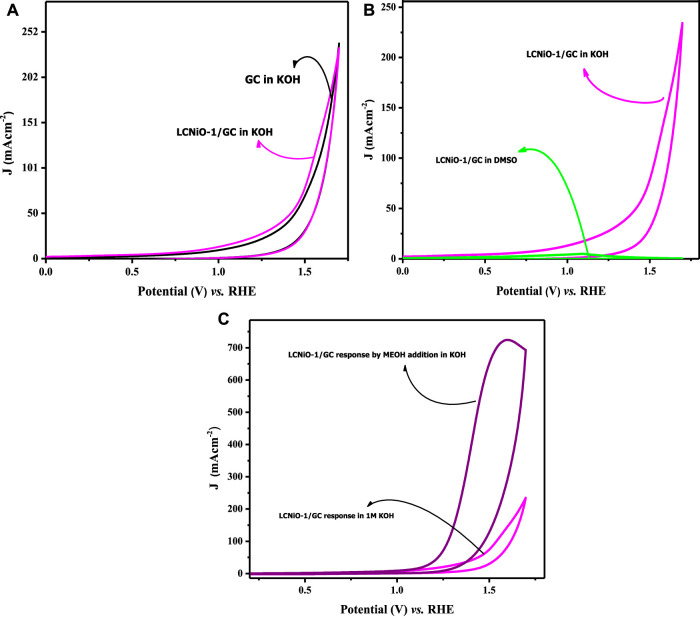
**(A)** Cyclic voltammetric response of OER for bare and LCNiO-1 modified GC electrode in KOH, **(B)** CV response in KOH (protic) and DMSO (aprotic) solvent at LCNiO-1 modified electrode, and **(C)** cyclic voltammogram for OER in KOH and methanol/KOH at LCNiO-1-modified GC.

Before analyzing the OER activity of electrodes, some pre-experimental verifications were performed to examine the intrinsic activity of the electrode in the KOH medium. Firstly, the response of the bare GC electrode was recorded in KOH electrolyte to confirm that there was no response of water-splitting peak by the GC itself in KOH. A comparison of CV profiles for bare GC and LCNiO-1-modified GC electrodes in KOH is given in Figure 6A. This comparison shows that the neat GC electrode scarcely responded (Kapałka et al., 2009), and a ∼0.7 A cm^−2^ peak current density for OER on the LCNiO-1-modified electrode confirmed the electroactive nature of these catalyst materials (Figure 6C). Hence, these voltammograms made it easier to decide whether to proceed with this material for water splitting.

Figure 6B represents the cyclic voltammograms of LCNiO-1-modified electrode in aqueous basic medium (i.e., in KOH) and in aprotic solvent (i.e., dimethyl sulphoxide; DMSO), which was recorded as a second verification step to counter-check whether the OER peak is solely due to the presence of water in the aqueous medium.

Peak current (density) is an essential criterion for checking the electroactivity of EC systems, and its absence from the voltammetric profiles may reflect the overall conditional EC process being infeasible. The conditions for such activity may also involve the solvent nature, in addition to many other factors like electrode surface roughness, surface area, and double-layer capacitance. A one-time CV test was performed in DMSO as an aprotic solvent for EC measurements to segregate any effects due to solvent and catalyst nature (Figure 6B). Voltammograms presented in Figure 6C showed no peak with LCNiO-1 modified GC electrode, while a small hump as a Faradaic activity at 1.59 V (*vs.* RHE) in KOH was observed, suggesting the electro-efficiency of the LCNiO material for the water oxidation reaction in the basic aqueous medium only.

#### OER responses in the presence of methanol

The OER in alkaline medium is given by Eq. 5a:

$${4OH}^{-}\to {2H}_{2}{O+4e}^{-}{+O}_{2}$$ (5a)

According to the OER universal mechanism proposed by Congling Hu and co-workers (Hu et al., 2019), the M-OH bond is formed initially by the oxidation of hydroxide ion adsorbed on the catalyst’s active site (**5b**; OH^−^ → OH^−^_ads_In the second step, the adsorbed metal hydroxide is converted into metal oxide (M-O) after proton-electron transfer through the paired reactions (**5c**; M + 2OH^−^_ads_ → M-O_ads_ + H_2_O + e-). Here, two M-O species either directly convert into oxygen and leave active sites or are transformed into M-OOH and then release oxygen after another proton-electron coupled reaction (**5d**; 2M-O → O_2_ + 2M).

In this context, OER responses were monitored in KOH electrolyte in the presence of methanol. The OER activity was multi-fold-enhanced, and CV profiles are presented in Figure 6C, rendering methanol as a strong facilitator for OER. Methanol forms a salt-like species MeO^−^K^+^ with KOH (MeOH + KOH → MeO^−^K^+^ + H^+^ + OH^−^), which decreases the diffusion of OH^−^ ions toward the electrode–electrolyte interface, and thus more OH^−^ ions adsorb on the surface of the catalyst, which could enhance the charge-transfer kinetics of the water oxidation process. This shows that methanol can act as a good facilitator for OER in the water splitting phenomenon, as it enhanced the small-current response in KOH to such a large peak current value with MeOH.

Hence, the above voltammograms ensure the electrocatalytic performance of the synthesized perovskite materials. Therefore, LaCe_1-x_Ni_x_O_3±δ_ was analyzed for water splitting, and the material with the best catalysis was identified based on various EC kinetic parameters evaluated using cyclic voltammetry.

#### Assessment of diffusion rates

**FIGURE 7:**
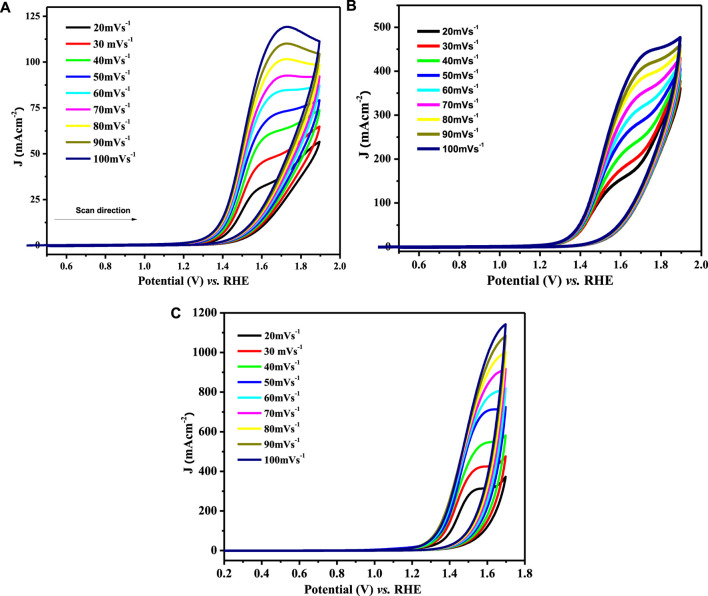
CV profiles for OER at **(A)** LCNiO-1/GCE, **(B)** LCNiO-5/GCE, and **(C)** LCNiO-9/GCE in 1 M methanol +1 M KOH at different scan rates (20–100 mV s^−1^).

The catalytic potential of a catalyst is scaled by its response to the redox process and its ease of facilitating the electron transfer process that directly relates to the diffusion coefficient values. The electroactivity of synthesized materials was investigated using CV experiments in 1 M KOH and 1 M methanol. Diffusion coefficients (D_o_) were derived from the recorded voltammograms by varying the scan rate (ν) from 20 to 100 mV s^−1^ within the selected potential window (0.2–1.8 V) at room temperature. The cyclic voltammograms for water oxidation observed at various scan rates are depicted in Figure 7.

For the determination of the diffusion coefficient, the Randles–Sevcik equation for the irreversible system was used:

$$I_{p}=\left({2.99\times 10}^{5}\right)n{\left(1-\alpha \right)}^{1/2}n_{\alpha }{AD}_{o}^{1/2}C\upsilon^{1/2}$$ (6)

In Eq. 6, I_p_ is the anodic peak current in ampere, n is the number of electrons involved in the reaction, n_α_ is the number of electrons involved in the rate-determining step, α is the transfer coefficient with a value lying in the range 0.3–0.7, A is the area of working electrode given as 0.07 cm^2^, D_o_ is the diffusion coefficient in cm^2^ s^−1^, C is the concentration given in mol cm^−3^, and υ is the scan rate in V s^−1^. The factor α (transfer coefficient) in Eq. 6 is calculated using Eq. 8, which signifies the charge transfer potential of the catalyst:

$$E_{pa}{-E}_{pa/2}=0.048\left(V\right)/\alpha n$$ (7)

Here, E_pa_ and E_pa/2_ are peak potential and half-peak potential values. For diffusion-controlled processes, the regression value must be above 0.5, as the slope value of the log form of Eq. 6. The linear dependence of the plot of ln I_pa_ versus ln ν (Supplementary Figure S3A) conforms to the diffusion-controlled behavior of the water electro-oxidation process at the LCNiO-9/GC electrode (Hegde, 2008), which is confirmed by the regression values of linear plots of I_p_ and υ^1/2^ (Supplementary Figure S3B). Fast or slow kinetics at the electrode surface are related to the mass transport coefficient. The mass transport coefficient for the present system was calculated by the following equation (Canales et al., 2015):

$$m_{t}{=D}_{o}\left(RT/F\upsilon \right)$$ (8)

The values of water oxidation diffusion and the mass transport coefficient are given in Supplementary Table S4, which describes the comparison of the water electrolytic responses of all the modified electrodes. The EC processes are mostly accompanied by a non-Faradaic and Faradaic region in I-E responses. The regions are distinguished based on their peak profiles. There is a minimal or only residual current in the non-Faradaic region, and the potential value at the advent of the Faradaic region (a steep rise in the current is observed) is termed the onset potential (E_on_). E_on_ has a significant role in the evaluation of the electrocatalytic performance of a material. Experimentally, all the potentials were scanned versus Ag/AgCl (E^o^ = 0.197 V) for electrochemical measurements. For comparison, this potential scale was then converted to RHE reference using the following equation:

$$E_{RHE}{=E}_{\left(Ag/Agcl\right)}{+E}_{\left(Ag/AgCl\right)}^{o}+0.059pH$$ (9)

**FIGURE 8:**
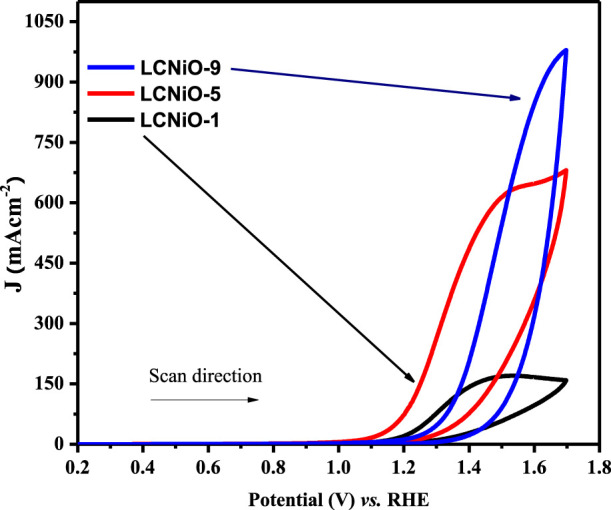
CV responses for OER on LCNiO-modified electrodes in KOH/MeOH system at 100 mV s^−1^.

The onset potential values for OER in 1 M KOH +1 M methanol follow the order 1.28 < 1.33 < 1.38 for LCNiO-1-, LCNiO-5-, and LCNiO-9-modified platforms, respectively. The comparative graph of CV responses of all compositions for OER at 100 mV s^−1^ is presented in Figure 10. The electrocatalytic trend in the potentials for OER studies varied in the order LCNiO-9 > LCNiO-5 > LCNiO-1. LCNiO-9/GCE responded with the highest peak current density and other electrochemical responses, due to its optimal doping level and its having the smallest average particle size. Therefore, the composition with x = 0.09 is best-suited for the OER. This is quite a promising finding in electrocatalysis and corresponds to the practical use of these simple perovskites as robust catalysts in OER and in overall water splitting (Figure 8).

**FIGURE 9:**
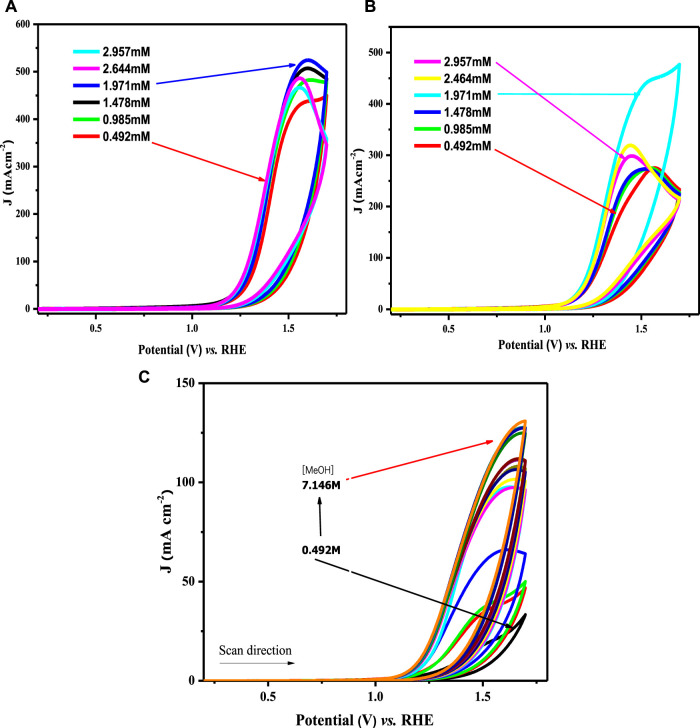
Cyclic voltammograms for water oxidation in different methanol concentrations on **(A)** LCNiO-1/GC, **(B)** LCNiO-5/GC, and **(C)** LCNiO-9/GC electrode.

To establish the appropriation of the prepared nanomaterials for the OER, the heterogeneous rate constant was estimated from the voltammetric responses of all modified electrodes using different methanol concentrations. Figure 9A illustrates the effect of changing methanol concentrations on the OER using a Nafion/LCNiO-1/GCE-modified electrode. An explicit trend of increasing peak current density with the increased methanol concentration up to an optimal value of saturation (i.e., 1 M) is demonstrated. A similar trend was also observed for the methanol concentration effect on OER using different modified electrodes, with results presented in Figures 9B,C.

All the modified electrodes showed similar voltammetric responses for OER, and amongst all compositions, the results for LCNiO-9 were found to be superior, representing multi-fold current density values with increasing methanol concentration. The catalytic propensity of the material can be checked by estimating the rate constant for the redox process, which is water oxidation in this case. A better value of the rate constant designates the reversible/irreversible nature or the ease of the process. The Reinmuth equation (Reinmuth, 1957) was used to estimate the rate constant for OER facilitated by the nanoceramics:

$${Ip=0.227nFACk}_{o}$$ (10)

**FIGURE 10:**
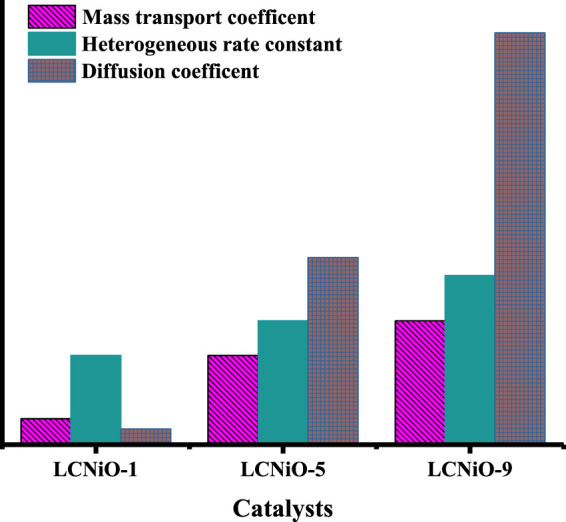
Comparison of different EC parameters obtained for all compositions.

Here, 0.227 is a constant term (various EC factors contribute to this value), I_p_ is the anodic peak current, F is Faraday’s constant, A is the area of the electrode, C is the concentration, and k_o_ is the heterogeneous rate constant. Reinmuth functional plots of all compositions confirm the diffusion-controlled process, as the values of regression coefficients lie in the range of 0.90–0.98 for all samples. The values of all kinetic parameters are collected in Supplementary Table S4 and presented in Figure 10.

Figure 10 suggests that the values of the heterogeneous rate constant for OER on all the modified electrodes were observed to be lower compared to mass transport coefficient values, suggesting the irreversible nature of the EC system under consideration. Moreover, LCNiO-9 nanocatalysts offered the highest values of D_o_, m_t_, and k_o_ for water electro-oxidation amongst all the modified electrodes.

**FIGURE 11:**
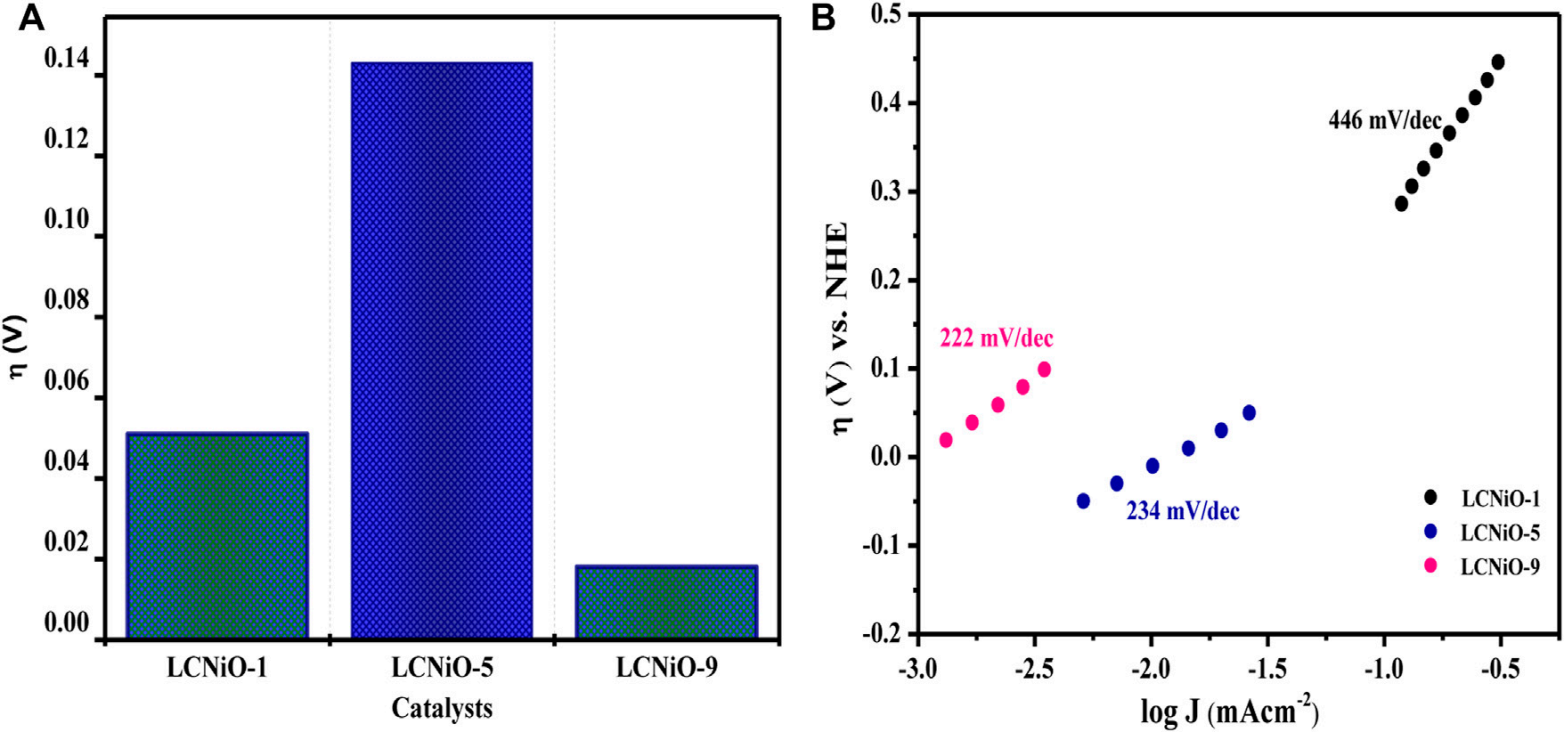
**(A)** Overpotentials for different LCNiOs observed at J value of 10 mAcm^−2^. **(B)** Tafel slopes determined for OER at 10 mV s^−1^.

Other OER parameters, including overpotential (η)—the difference between the thermodynamic half-reaction potential and the potential (E_on_) at which the charge transfer process occurs—for all compositions was determined to be 10 mA cm^−2^ current density, as presented in Figure 11A, with the lowest overpotential value for LCNiO-9 nanomaterials.

**FIGURE 12:**
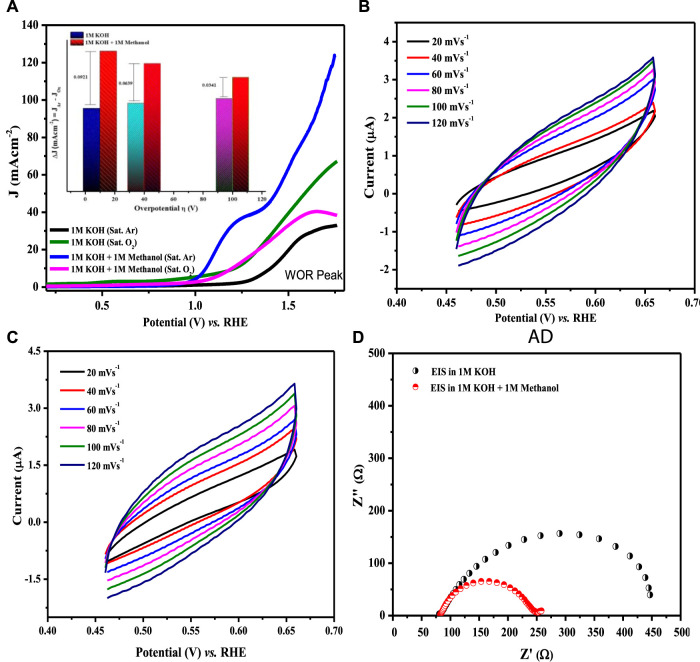
**(A)** LSV scans for water oxidation at 20 mV s^−1^ in KOH, KOH/methanol mixture in oxygen- and argon-saturated environments using LCNiO-9-modified GC. The inset presents the difference of current densities in argon- and oxygen-saturated environments. **(B,C)** CV plots at different sweep rates in the KOH and KOH/methanol system. **(D)** EIS spectra at a voltage (@10 mA cm^−2^) in KOH and KOH/methanol.

Current-overpotential plots (Tafel plots) are used to further assess the kinetics and mechanism of OER. The anodic polarization curves for OER were recorded in 1 M KOH and the optimal concentration of methanol at the scan rate of 10 mV s^−1^. Tafel slopes were determined using the Tafel equation (Bard and Faulkner, 2001):

η=a+b⁡log⁡j (11)

where a is the Tafel constant measured in volts, b is the Tafel slope in mV dec^−1^, and J is the current density in mA cm^−2^. Tafel slopes for all compositions are presented in Figure 11B. The maximum value of the Tafel slope was observed for LCNiO-1 (i.e., 446 mV dec^−1^), and the minimum value was 222 mV dec^−1^ for LCNiO-9; thus, LCNiO-9 showed better performance for the OER under similar conditions. The best composition, LCNiO-9, was further evaluated for its electrocatalytic potential for alkaline OER using linear sweep voltammetry, electrochemical impedance spectroscopy, and chronoamperometry. The same working electrode was used for electrochemical testing, and all measurements were taken in a three-electrode assembly under an argon blanket. LSV scans were recorded in KOH and KOH/methanol mixed media under the oxygen and argon blankets in the potential scan range of 0.2–1.8 V at 20 mV s^−1^, as illustrated in Figure 12A. These scans demonstrate a sharp negative shift in OER oxidation potential values in the KOH/methanol mixed medium under an Ar-saturated environment, in comparison to KOH medium only and oxygen saturation; therefore, all further analysis and tests were conducted under argon saturation. A prominent rise in the OER peak current densities was also observed in the KOH/methanol mixed medium as compared to KOH medium. The difference in current densities under both gaseous blankets, ΔJ (J_sat. argon_–J_sat. oxygen_) was also analyzed in both media at η_10_, η_40_, and η_100_ (the overpotential values at 10, 40, and 100 mA cm^−2^ current densities for OER) and is presented in the inset of Figure 12A. Increased current densities in KOH/methanol medium suggested choosing the mixed solvent system in further OER studies.

Cyclic voltammetry in the non-Faradaic region with different scan rates was also performed in both the KOH and KOH/methanol media to determine the double-layer capacitance, C_dl_, using a linear relationship between scan rate and the difference of current densities (ΔJ = J_a_ − J_c_) at 0.55 V, as presented in Figures 12B,C. C_dl_ values estimated from the linear slopes were 11.18 and 11.82 μF cm^−2^ in KOH and KOH/methanol medium, respectively (Supplementary Figure S4). The slight increase in C_dl_ value in the mixed solvent suggests enhanced energy density. Thus, improved OER performance in the presence of methanol (as C_dl_) is directly related to the electrochemically active surface area. The higher the active surface area, the better the electrocatalytic activity.

EIS analysis (Figure 12D) was performed at a potential at which the current density approached 10 mA cm^−2^ to investigate the detailed electrochemical process. Charge transfer resistance (R_ct_) was determined by model-fitting the EIS spectrum using Randle’s model (Egelund et al., 2016), which is 109 Ω in KOH and 57 Ω in the mixed-solvent system. A significant decrease in R_ct_ resistance with the addition of methanol infers the facility of the diffusion phenomenon due to the formation of salt-like species, M-OOH (Eq. 6), and therefore validates the enhancement of OER performance with the methanol addition.

Good activity and long-term stability are the two major concerns in the catalyst’s design for practical applications (Ashraf et al., 2022). Therefore, a durability test was performed using an LCNiO-9-modified GC platform *via* chronoamperometry at η_10_ in 1 M KOH, with 1 M methanol for 50 h. No obvious degradation was observed in current densities during the chronoamperometric scans, as presented in Supplementary Figure S5.

All these results further confirm that methanol addition in KOH enhances the water oxidation process by providing further adsorbed species for enhanced electrocatalysis (Hu et al., 2019). LCNiO-9 composition could be utilized as an OER electrocatalyst for commercial and long-term applications.

A comparison of the OER parameters of the LCNiO/GC-modified electrode with literature-reported data is presented in Table 3. Table 3 illustrates the enhanced OER performance, in terms of low onset and overpotential values, over LCNiO-modified GC electrodes in comparison to other catalysts. The improved activity is attributed to the small crystallite sizes of the catalysts produced by the co-precipitation route, which provide more active sites for catalysis. The addition of methanol in 1 M KOH as an OER-facilitating agent significantly upgrades the OER performance of LCNOs/GC.

**TABLE 3:** Comparison of OER performance with literature reported data.

Catalyst	E_onset_ (V)	η_10_ (mV)	References
^a^IrO_2_	-	341	Lee et al. (2012)
^a^RuO_2_	1.55	505	Anand et al. (2021)
^a^LaFeO_3-δ_	1.57	437	Nguyen et al. (2021)
^a^LaFeO_3-δ_	1.64	510	Gao et al. (2020)
^a^Sm_0.5_Sr_0.5_FeO_3−*δ*_	1.52	369	She et al. (2018)
^a^LaCoPrO_3_	1.52	312	Xie et al. (2021)
^a^LaCoO_3_	1.55	326	Lu et al. (2018)
^a^LaFeO_3_	1.41	420	Dai et al. (2019)
^a^La_0.5_Sr_0.5_Ni_0.4_Fe_0.6_O_3−*δ*_	1.40	330	Cheng et al. (2018)
^a^LaCo_0.8_V_0.2_O_3_	1.30	268	Sun et al. (2020)
^a^LaCo_0.9_Ni_0.1_O_3_	1.57	650	Wang et al. (2019)
LaCe_0.99_Ni_0.01_O_3_	1.28	51	This work
LaCe_0.95_Ni_0.05_O_3_	1.33	143	This work
LaCe_0.91_Ni_0.09_O_3_	1.38	18	This work

^a^ Electrolyte: 1M KOH; substrate: GCE.

## Conclusion

Nickel-doped lanthanum cerate, LCNiO (LaCe_1-x_Ni_x_O_3±δ_, where x = 0.01, 0.05, and 0.09) nanomaterials were successfully synthesized using an ammonia-precipitation method and were verified for the positive impact of doped amount on the resultant structural and electrochemical properties of the parent LCO perovskite. The synthesized nanoceramics were used as catalyst materials for water electro-oxidation using CV, and the conductive behavior of materials was inferred from EIS measurements. All materials were found to possess good conductive behavior, with the best outcomes observed for LCNiO-9 with 9% doped nickel in place of cerium, resulting in a high value of diffusion coefficient (3.65 × 10^–5^ cm^2^s^−1^) for water electro-oxidation. The small crystallite size of the LCNiO-9 composition (i.e., 18 nm) and the active surface area of 0.055 cm^2^ corresponded well with the current density of 900 mA cm^−2^ and a low value of charge transfer resistance, 28.92 Ω. The heterogeneous rate-constant value was observed to be 1.5 × 10^–4^ cm s^−1^, a decisive factor in the diffusion-controlled nature of the process. Stability tests also confirmed the long-term application of the prepared electrodes. The use of mixed media as KOH/methanol for the OER process is reported here for the first time. Herein, methanol promoted the OER activity to an explicit limit. Many orders increment in the current density, and a substantial negative shift in the peak potential for OER was observed using the LCNiO-catalyst-modified electrodes in the mixed medium. The prolific activity can be attributed to the oxidized species and the involvement of the interstitial oxygen anions such as MOOH^−^ or other thermodynamically favored intermediates. Fabbri and co-workers have also detailed the mechanistic possibilities of OER in alkaline and other media, for which the possible pathways are presented in Figures 2, 3 involving such oxygen resource species (Kapałka et al., 2009; Prakash et al., 2017). Methanol oxidation is usually observed in a potential window of 0.4–0.8 V, with a crossover potential peak in a lower potential range. Such phenomena have been referred to by many studies (Djoudi et al., 2012; Liu et al., 2021). Accordingly, the OER reaction does not overlap with the methanol crossover oxidation range, and it is only mediated by the formation of oxygen-rich species by coupling with the metal oxide matrix in a low thermodynamic pathway to facilitate the OER (Reinmuth, 1957; Bard and Faulkner, 2001; Hegde, 2008; Canales et al., 2015; Hu et al., 2019). The investigation of LaCeNiO_3_ perovskites was extended to high-temperature fuel cells for all compositions integrated as cathode catalytic materials. The lowest activation energy and bandgap was observed for devices using LCNiO-9 electrodes. Henceforth, LCNiO-9 demonstrated better electrocatalytic performance because of its optimal loading, its porous structure with high surface area, and its comparatively small particle size, tuning the electronic structure for enhanced performance in solid state applications at high temperatures.

In all, the entirely different energy domains of OER and SOFC are capacitated by the same material that defines the structural correlation with the defects created by Ni-doping and the resultant vacancies in the intrinsic microstructure. Robust water electro-oxidation and promising solid-state high-temperature EIS performance validated the LaCeNiO_3_ materials as good electrocatalysts for oxygen production and oxygen reduction reactions in energy devices, respectively.

## Data Availability

The original contributions presented in the study are included in the article/Supplementary Material. Further inquiries can be directed to the corresponding author.
