# Supplementary material for: Nickel-doped lanthanum cerate nanomaterials as highly active electrocatalysts
Source: Front Chem. 2022 Nov 22;10:1064906. doi: 10.3389/fchem.2022.1064906 (PMC9731227; doi:10.3389/fchem.2022.1064906)
Supplement: Supplementary file 1 [file DataSheet1.PDF]

Nickel-Doped Lanthanum Cerate Nanomaterials as Highly Active Electrocatalysts

Supplementary Data

Table S1. Sample codes with their molecular formula for LCNiO compositions.

Sample code	Molecular Formula	Mol % of Ni
LCO	$LaCeO_3$	0.00
LCNiO-1	$LaCe_{0.99}Ni_{0.01}O_{3\pm\delta}$	0.01
LCNiO-5	$LaCe_{0.95}Ni_{0.05}O_{3\pm\delta}$	0.05
LCNiO-9	$LaCe_{0.91}Ni_{0.09}O_{3\pm\delta}$	0.09

Table S2. Crystallite sizes and estimated active surface area of the synthesized LCNiOs.

Sample codes	Crystallite size, D_{av} (nm)	Active surface area (cm^2)
LCNiO-1	28	0.011
LCNiO-5	22	0.012
LCNiO-9	18	0.055

Table S3. wt.% Elemental composition from EDX analysis of LCNiOs.

Samples	wt. % of O	wt. % of La	wt. % of Ce	wt.% of Ni
LCNiO-1	21.51	55.56	22.89	0.04
LCNiO-5	22.07	52.62	24.35	0.96
LCNiO-9	23.47	52.16	23.32	1.05

Table S4. Estimated values of diffusion and mass transport coefficients derived from the CV and EIS data.

Samples	Diffusion coefficient D_o/ 10^{-5} cm^2 s^{-1}	Mass transport coefficient $m_t/10^{-2}$ cm s^{-1}	Heterogenous rate constant, $k_o/10^{-3}$ cm s^{-1}
LCNiO-1	0.14	0.23	0.079
LCNiO-5	1.66	0.79	0.110
LCNiO-9	3.65	1.10	0.150

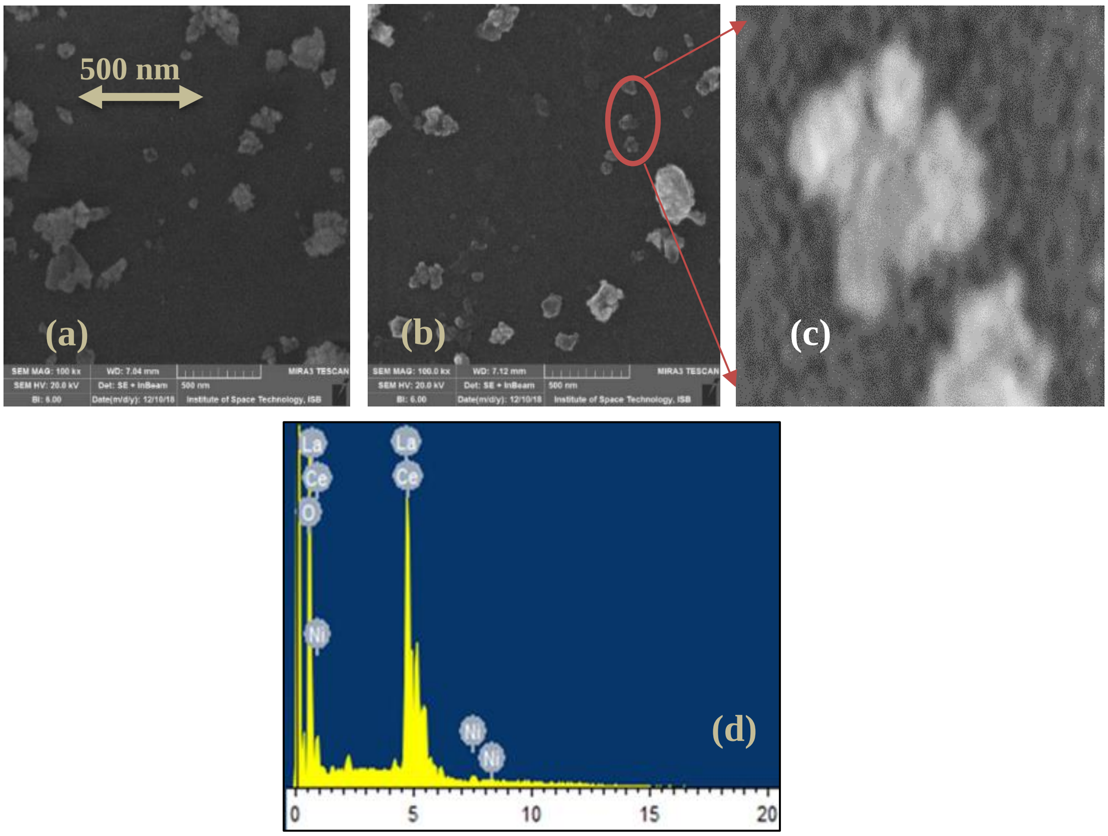

Figure S1. Microstructures observed for $LaCe_{1-x}Ni_xO_{3\pm\delta}$ (a & b; x= 0.01 and 0.05) at 500 nm resolution and magnified image b for homogeneous particle size (c) using SEM, EDX spectrum for LCNiO-9 (d).

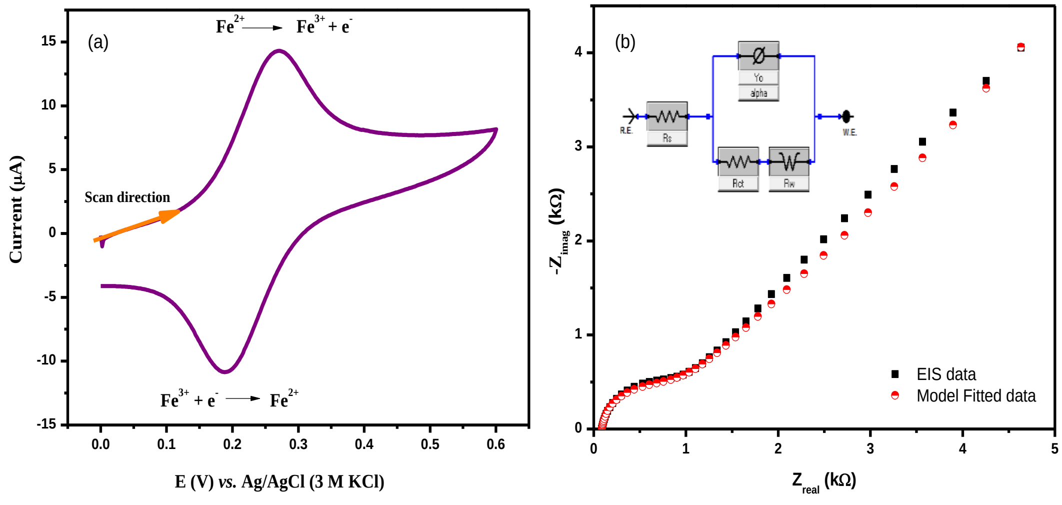

Figure S2. (a) Cyclic voltammetric responses of LCNiO-9/GC electrode in 5 mM potassium ferrocyanide and 1 M KCl at 0.1 Vs^{-1} and 298 K (b) Nyquist plot for 5 mM $K_4[Fe(CN)_6]$ and 1 M KCl on LCNiO-9 modified GC electrodes with equivalent circuit model in the inset.

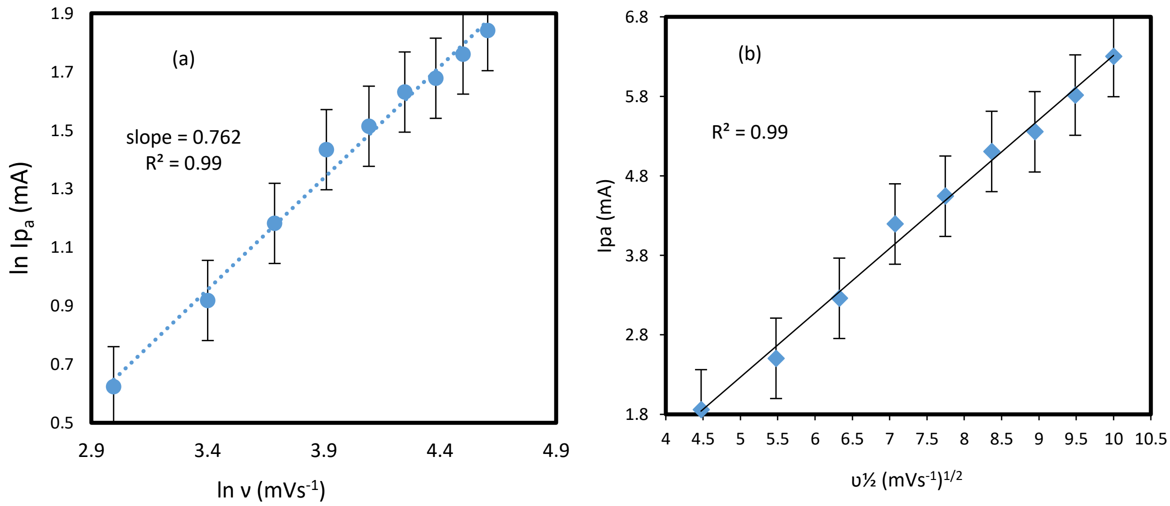

Figure S3. (a) Linear functional plot for determination of diffusion coefficient value for LCNiO-9 (b) Logarithmic plot of peak current and scan rate for LCNiO-9.

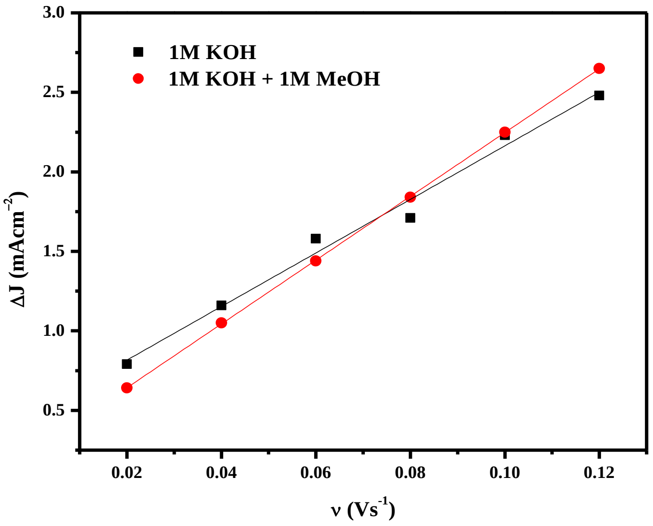

Figure S4. Linear plot of ν and ΔJ for the calculation of C_{dl} using LCNiO-9 modified GC platforms.

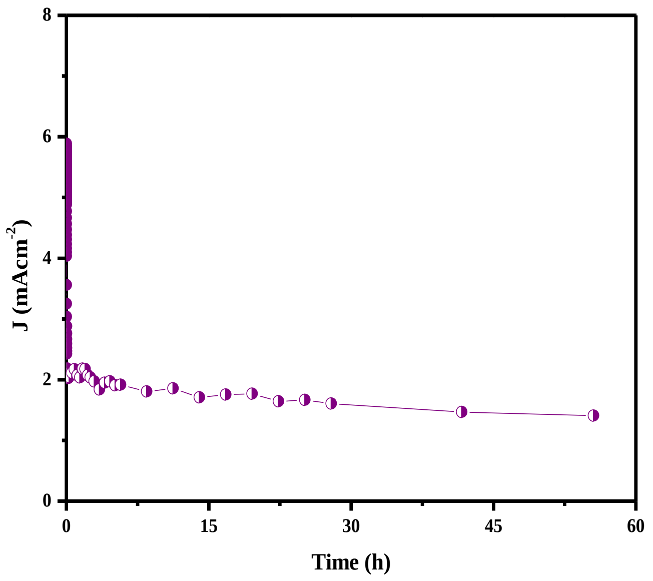

Figure S5. Chronoamperometric scan performed for 50 h at a voltage (@10 mA cm^{-2}) using LCNiO-9 modified GC platform in 1 M KOH/1 M methanol.
